# No apparent effect of a magnetic pulse on free-flight behaviour in northern wheatears (*Oenanthe oenanthe*) at a stopover site

**DOI:** 10.1098/rsif.2021.0805

**Published:** 2022-02-16

**Authors:** Thiemo Karwinkel, Michael Winklhofer, Paula Christoph, Dario Allenstein, Ommo Hüppop, Vera Brust, Franz Bairlein, Heiko Schmaljohann

**Affiliations:** ^1^ Institute of Avian Research ‘Vogelwarte Helgoland’, An der Vogelwarte 21, 26386 Wilhelmshaven, Germany; ^2^ Institute for Biology and Environmental Sciences (IBU), Carl von Ossietzky University of Oldenburg, Carl-von-Ossietzky-Straße 9-11, 26129 Oldenburg, Germany; ^3^ Research Center for Neurosensory Sciences, Carl von Ossietzky University Oldenburg, 26111 Oldenburg, Germany; ^4^ Max Planck Institute of Animal Behavior, Am Obstberg 1, 78315 Radolfzell, Germany

**Keywords:** bird migration, navigation, magnetoreception, magnetic-particle-based sensor, magnetic pulse, geomagnetic map

## Abstract

Naïve migrants reach their wintering grounds following a clock-and-compass strategy. During these inaugural migrations, birds internalise, among others, cues from the Earth's magnetic field to create a geomagnetic map, with which they navigate to destinations familiar to them on subsequent migrations. Geomagnetic map cues are thought to be sensed by a magnetic-particle-based receptor, which can be specifically affected by a magnetic pulse. Indeed, the orientation of experienced but not naïve birds was compromised after magnetic pulsing, indicating geomagnetic map use. Little is known about the importance of this putative magnetoreceptor for navigation and decision-making in free-flying migrants. Therefore, we studied in unprecedented detail how a magnetic pulse would affect departure probability, nocturnal departure timing, departure direction and consistency in flight direction over 50–100 km in experienced and naïve long-distant migrant songbirds using a large-scale radio-tracking system. Contrary to our expectations and despite a high sample size (*n*_total_ = 137) for a free-flight study, we found no significant after-effect of the magnetic pulse on the migratory traits, suggesting the geomagnetic map is not essential for the intermediate autumn migration phase. These findings warrant re-thinking about perception and use of geomagnetic maps for migratory decisions within a sensory and ecological context.

## 1. Introduction

Migratory songbirds possess the fascinating ability to return to previously used breeding or wintering locations with a precision of centimetres, despite migrating over distances of up to tens of thousands of kilometres [1]. This ability is resilient even against natural translocations, e.g. through wind [2], or anthropogenic translocations to unknown areas [3]. To compensate for those, birds need to determine their actual location in relation to their goal. Although compass orientation mechanisms [4] provide seasonally appropriate directional information, they are generally not sufficient to determine a location. Instead, a navigational map is required for ‘true navigation' [5].

This cognitive map is not inherited, but instead must be learned during the inaugural migration, as when juvenile songbirds follow a genetically encoded direction and timing programme, the so-called clock-and-compass orientation [6,7]. During the subsequent return migration, they integrate learned map information to navigate back to their natal/breeding location [8–10]. The Earth's magnetic field has been hypothesised to be used as a map factor [5,11–14] since it varies systematically across the globe. In previous experiments testing for true geomagnetic navigation, caged animals were virtually displaced, i.e. exposed to magnetic field parameters mimicking a location which would trigger a compensatory directional behaviour if true navigation, distinct from compass orientation, was exhibited [12,15]. The observation that virtually displaced test animals oriented as if they had been physically relocated can be taken as evidence for geomagnetic map navigation [12,15,16], but see [17].

Despite these advances in understanding geomagnetic navigation [18], the underlying sensory structures remain an unsolved mystery in sensory biology. The magnetite hypothesis [19] posits the existence of specialised sensory cells containing magnetic particles, following the example of magnetotactic bacteria, which have intracellular, membrane-enclosed magnetic particles (magnetosomes) [20]. Magnetotactic bacteria behave similarly to a physical ‘compass needle', which can be reversed after exposure to a brief but strong magnetic pulse [21]. Therefore, the key experiment to test for the involvement of magnetic particles consists of a pre-treatment with a magnetic pulse aimed at manipulating the sensor cells [22]. Indeed, songbirds tested in Emlen funnels [23–28] had deflected orientations after pulsing, but were still oriented at the group level, which suggests that the ability of magnetic sensing was not lost. No pulse effect was observable when the beak had been anaesthetised before pulse treatment [29,30] or when the bird was on inaugural migration [31,32]. The pulse effect wanes off within *ca* 10 days following the application [23,33]. Because the sensor involved in magnetic compass orientation, i.e. the putative radical-pair-based sensor [34], does not seem to be affected by a magnetic pulse pre-treatment (no effect on young birds), it has been argued that the magnetic-particle-based sensor is exclusively involved in geomagnetic map navigation, but not in magnetic compass orientation [27].

All these aforementioned studies were aimed at assessing the involvement of magnetic particles in magnetoreception and were therefore conducted in a controlled artificial laboratory environment, with caged birds having no access to non-magnetic cues. This prompts the question of whether the observed behaviour, particularly the deflected directional response, would also occur under natural conditions, where a number of environmental stimuli, e.g. visual, olfactory and landscape cues, feed into a multisensory, multi-cue navigational map [35]. Studies on birds in free-flight [36–38] have great potential to address this question, but recording behavioural traits of small night-migratory songbirds in the wild is technically challenging. Holland [36] and Holland & Helm [37] overcame these problems by radio-tracking wild songbirds pre-exposed to a magnetic pulse and confirmed the findings of the behavioural laboratory experiments: adult, i.e. experienced birds that have successfully mastered at least one full migratory journey, reed warblers (*Acrocephalus scirpaceus*) and European robins (*Erithacus rubecula*) but not juvenile, i.e. naïve, European robins were deflected in their initial departure direction by a magnetic pulse; the effect in adults decreased or was absent after a period of about 10 days [37]. Additionally, Holland *et al*. [38] tracked catbirds (*Dumetella carolinensis*) over a large scale (greater than 50 km) after magnetic pulsing, but no effect of the pulse was expected to be found, as most birds departed after the critical period of 10 days. Despite these important results, assessing the effects of a magnetic pulse on further migratory traits, like departure probability, departure timing within the night, initial departure direction and the consistency of this initial flight direction after departure over tens of kilometres, remains a major challenge to understanding navigation in migratory birds.

To fill parts of these gaps in knowledge, we caught migrating adult and juvenile northern wheatears (*Oenanthe oenanthe*, hereafter wheatear), a long-distance night-migratory songbird [39,40], on the small remote island of Helgoland in the German Bight during autumn. Helgoland serves as a stopover, where migrants recover from the previous migratory flight, rest and fuel to prepare for the upcoming flight. In order to evaluate the possible effects of the magnetic pulse on the specific migration properties, we applied the following experimental approach. We temporarily caged birds, pulsed these with a high urge to migrate 6 h before sunset, i.e. before they make their daily departure decisions [41], attached radio-tags and released them on days with weather conditions favourable for migration. The local [42] and the German Bight-encompassing array [43] of digital radio-receiving stations (Motus Wildlife Tracking System, [44]) automatically recorded the response to the treatment. This magnetic pulse experiment with free-flying birds controlled for the first time, to our knowledge, in unprecedented detail, ‘*over the consistency of physiological state and of environmental cues*' [37, p. 3] between experimental and control groups. First, we assessed the potential effect of this treatment on the bird's departure probability, i.e. the daily decision to resume migration. Second, we studied its potential effect on the timing of departure within the night. This is an important trait because it affects the duration, and thus distance, of the nocturnal migratory endurance flight [45]. Third, we analysed whether the magnetic pulse affected the initial departure direction as in former studies [36,37]. Fourth, we considered the flight consistency across the German Bight to assess whether birds may have changed their directional decision for the first 50–100 km off Helgoland in response to the magnetic pulse. Based on current knowledge, we predicted that the magnetic pulse affects all four migratory traits with delayed departure decisions and deflected or more scattered directions in adult, but not juvenile, wheatears.

## 2. Methods

### 2.1. Site and species

**Figure 1.**
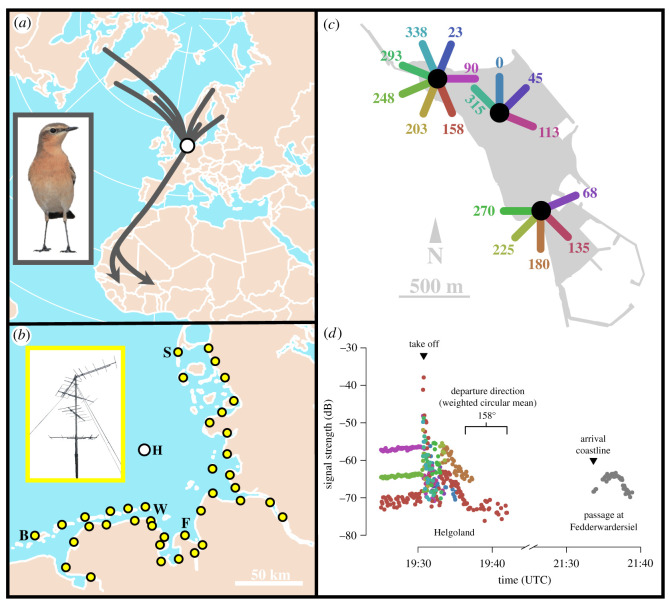
(*a*) Estimated autumn migration routes of northern wheatears (*Oenanthe oenanthe*) passing the island of Helgoland (white dot). (*b*–*d*) Free-flight behaviour was recorded using radio telemetry: (*b*) Locations of automated radio-receiving stations on Helgoland (H) and along the coastline of the German Bight (yellow dots). The closest station to Helgoland is Wangerooge (W) in the south, about 44 km away. The radio-receiving stations on Sylt (S, about 72 km from Helgoland) and Borkum (B, about 104 km from Helgoland) encompass the remaining stations. Inset shows antennas of an example radio-receiving station. (*c*) On Helgoland, three automated radio-receiving locations with 16 antennas, evenly spaced about 22.5° apart in direction, are installed to precisely determine departure timing and direction from Helgoland. (*d*) An example of a nocturnal departure event as recorded by our system displaying signal strength over time (coordinated universal time: UTC). Colours correspond to antenna directions on Helgoland in (*c*). The plot starts with three parallel lines, indicating the bird being stationary. The following peak with increasing numbers of different antennas (colours) indicates the take-off. The decreasing number of antennas and signal intensity indicates the bird flying off the island in a specific direction until the signal is lost (see the electronic supplementary material for details). The grey dots after approximately 2 h indicate the passage at the coastline radio-receiving station in Fedderwardersiel (F in (*b*)), with the first detection defined as coastline arrival. Photos by T.K.

The experiment took place on Helgoland (54°11′ N, 07°53′ E), a small island in the German North Sea about 50 km off the coast (figure 1*a–c*). During autumn migration, wheatears of two subspecies are present on the island from the end of July to mid-October [46]. We caught first-calendar year (hereafter juvenile) and older than first-calendar year (hereafter adult) wheatears with mealworm-baited spring traps from 28 August to 18 September 2020. Birds were ringed, aged and sexed according to Svensson [47]. The maximum wing chord was measured to the nearest 0.5 mm. Wheatears breed only occasionally in single years on Helgoland [46] with no breeding records in 2020 [48], so all caught wheatears were true passage migrants.

### 2.2. Experimental procedure

**Figure 2.**
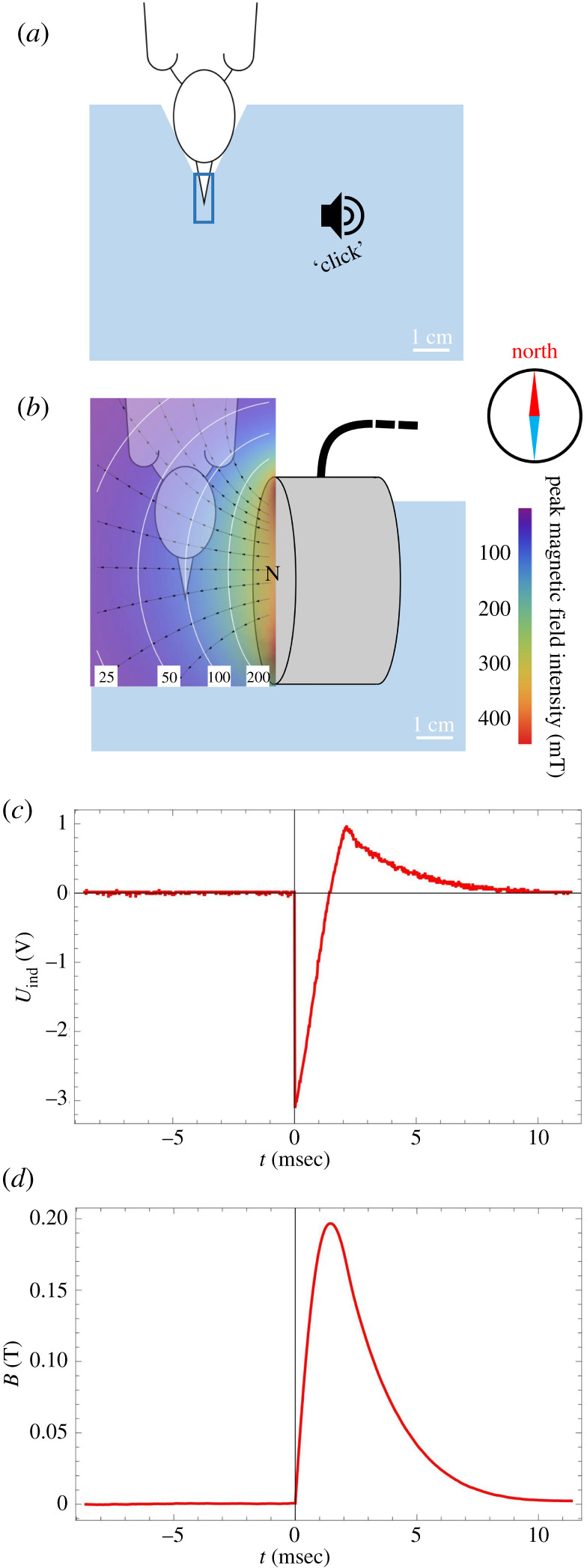
Application of the control (*a*) and experimental treatment (*b*) to the birds. Birds were hand-held and the head was placed into an indentation of a foam block (light blue box, extruded polystyrene foam, XPS) fixed to a wooden table. The beak of the bird was immobilised by positioning it into a small piece of plastic tube (dark blue) in the foam. (*a*) The control group only experienced a short ‘click' sound, but all handling was similar to the experimental group. (*b*) The experimental group experienced a magnetic pulse from a small coil (grey cylinder; 50 mm diameter with 15 × 15 windings of 1 mm copper wire) with the coil's magnetic north pointing perpendicular to the beak. The distance between the beak and the coil was adjusted to yield a peak magnetic field of *ca* 0.1 T (100 mT). Heat map shows peak magnetic field intensity with isolines in white and selected magnetic field lines in black. All experiments were performed with the birds directed southwards (south anterior). (*c* + *d*) Time course of the magnetic field intensity of the magnetic pulse. The measurement was taken with a self-made pick-up induction coil (5 cm diameter, six turns) placed on the pulse coil and connected to an oscilloscope (Tektronix MDO3032, Beaverton USA). The induced voltage measured (*c*), which is proportional to the rate of change of the magnetic field with respect to time, was integrated to yield the time course of the magnetic field in Tesla (*d*). After triggering the pulse at *t* = 0, the magnetic field reaches its maximum at 1.5 ms to then decay within 8 ms.

Immediately after catching, birds were housed indoors for a few days in individual plastic cages (40 × 30 cm, 40 cm high) with *ad libitum* food (mealworms, *Tenebrio molitor*) and water for accumulating fat, i.e. fuel, at the island station of the Institute of Avian Research ‘Vogelwarte Helgoland'. Wheatears showed no indication of stress under such conditions in another study [49]. For the experiment, we chose only days with migration-favourable weather conditions (no rain, wind speed less than 8 m s^−1^) [50,51]. We assigned equal numbers of housed adult and juvenile birds to the control or experimental group. To calculate exact fuel load, birds were weighed to the nearest 0.1 g and muscle was scored before the experiment [52]. We found no differences between control and experimental groups in fuel load, subspecies, sex, day of year of experiment, cloud cover and wind assistance in the night after release, which are known to affect the migratory traits of interest in wheatears [51,53] (electronic supplementary material, table S1). We applied the magnetic pulse to the birds outdoors on a wooden table, where the coil of the magnetic pulser (‘Beck-Pulser', magnetic pulse generator, Ing. Büro L. Albrecht, Heist, Germany) was fixed into a foam block (figure 2*a,b*). We checked the functionality of the pulser and its characteristic intensity of 0.1 T (100 mT) on every experimental day with a magnetometer (Gaußmeter HGM09s, MAGSYS Magnet Systeme GmbH, Dortmund, Germany). The magnetic field lines of the pulse were perpendicular to the beak, the latter pointing south anterior (figure 2*b*). The magnetic field rises within *ca* 1.5 ms to its peak value and decays within 8 ms (figure 2*c*,*d*). For the control group, we constructed a similar foam block, but the birds experienced just a ‘click' sound (figure 2*a*), comparable to the one produced by the pulser, created by tapping a finger on the foam block, as our coil did not allow an opposite, self-cancelling current as did the double-wrapped coil in [36]. Immediately after the pulse or the sham treatment, every bird was equipped with a radio transmitter (see details below) and released. The release time was about 6 h before sunset.

### 2.3. Radio-tracking

We attached uniquely coded radio transmitters (NTQB-2, Lotek Wireless Inc., Canada; burst interval between 2.3 and 5.3 s) to the birds using individually adjusted leg-loop harnesses [54]. The total weight of transmitter and harness was maximum 0.35 g, which did not exceed 1.6% of the bird's body mass (median: 1.2%) and is therefore well below the 3–5% recommended threshold for attached devices on birds [55]. We used an automated digital radio-telemetry system consisting of four SensorGnome receivers (www.sensorgnome.org) and equipped with 16 radially aligned antennas (six-element Yagi antennas, Vårgårda Radio AB, Sweden) located at three sites on Helgoland (figure 1*c*). Thirty-nine comparable radio-receiving stations are established along the German coastline and on coastal islands (figure 1*b*) [43]. This large-scale system continuously received radio signals on a used frequency (here 150.1 MHz) during the study period to track the wheatears' departure events from Helgoland and their subsequent movements across the German Bight. All stations are part of the Motus Wildlife Tracking System; see http://www.motus.org and [44]. From the radio-tracking data, as received from Motus [44], we determined departure date and timing within the night, and departure direction and passage at the coastline (as shown in figure 1*d*), using an algorithm written by the authors, in a replicable and double-blind approach to avoid any observer bias [42,51]. Further details about how the radio-tracking data was analysed is given in figure 1 and in the electronic supplementary material.

### 2.4. Statistics

All statistical analyses were performed using R v.4.0.3 statistical software [56]. We calculated fuel load on the day of the experiment based on wing length, weight and muscle score after Kelsey *et al*. [57]. Precipitation (mm) and cloud cover (eighth) in the night after release at 143 min after sunset, as derived from a former study with wheatears on Helgoland [51], were provided by the local weather station (German Weather Service, DWD). After Kemp *et al*. [58], wind assistance (m s^−^^1^) for a direction of 176°, as derived from a former study with wheatears on Helgoland [51], was calculated using NCEP reanalysis data [59] (National Oceanic and Atmospheric Administration (NOAA); Boulder, CO, USA; http://www.cdc.noaa.gov/cdc/data.ncep.reanalysis.derived.html), using the same approach as in Müller *et al*. [42]. Geographical data for maps were downloaded from the GSHHG database of the NOAA (https://www.ngdc.noaa.gov/mgg/shorelines/) [60].

To assess whether our treatment affected the birds' departure probability, we ran a generalised linear model including wind assistance (m s^−1^; continuous), cloud cover (eighth; categorical), day of year (Julian Day, first January = 1; continuous), fuel load (relative to bird's lean body mass, continuous) (all parameters z-transformed), treatment condition (experiment/control; categorical) and age (adult/juvenile; categorical). Because model assumptions were violated and we found no solution to overcome this, we used chi-square tests to assess potential differences in the departure probability between the control and experimental groups for both age classes.

To explain variation in departure timing within the night (represented as minutes after sunset; continuous), we used a linear model including the abovementioned parameters and all two-way interactions between them and the treatment group. We excluded the parameter cloud cover owing to positive collinearity with wind assistance and day of year (VIF > 50, [61]). Because no two-way interaction was significant, all were excluded from the final model. One juvenile control and one adult experimental bird departed before sunset (−167 and −235 min after sunset, respectively), but we found no reason to exclude those birds from the analysis. The residual analyses did not indicate a violation of model assumptions.

To assess the circular variables (departure direction, consistency in flight direction), we applied circular statistics using the R packages ‘CircStats' [62] and ‘circular' [63]. Our directional data were grouped to a certain extent (see section Radio-tracking). Because the Mardia–Watson–Wheeler test [63] randomly breaks such groupings (ties) apart, we repeated the test 10 000 times to exclude any bias owing to random tie-breaking and then provided the median of the test parameters (see R-code in the electronic supplementary material for details).

The effect of the magnetic pulse is assumed to last for several (up to 10) days [28,37]. Because all our birds left the island within 5 days after the pulse application, we included all birds in the analyses and did not assess whether the potential effect weakened over time. However, as we did not know whether the effect duration would be as long in wheatears as in the other species, i.e. European robins [37] and silvereyes (*Zosterops lateralis*) [23,33], we additionally repeated all analyses using only birds that departed in the first night after the pulse application. As there were no differences in the results between these two approaches, we provide all results considering only birds departing in the first night in the electronic supplementary material. The full R-code is available in the electronic supplementary material.

## 3. Results

### 3.1. Departure probability

**Figure 3.**
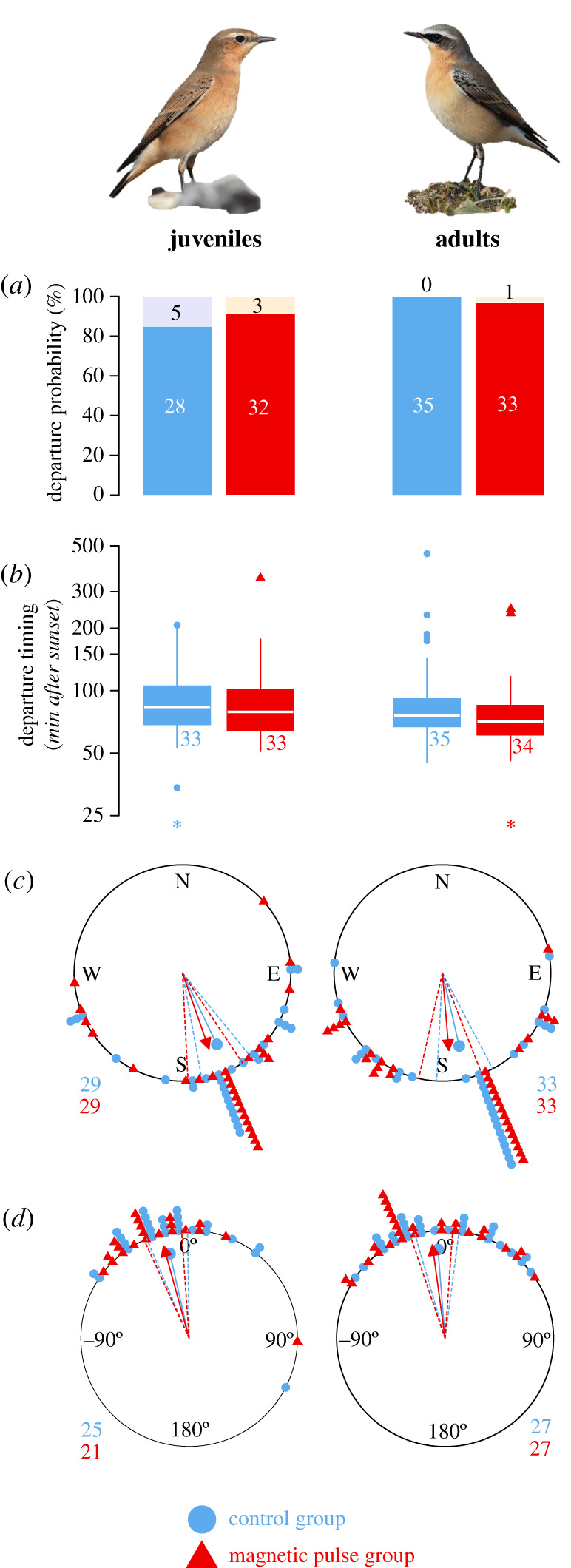
Free-flying migratory behaviour of juvenile (left column) and adult (right column) northern wheatears (*Oenanthe oenanthe)* after treatment with a magnetic pulse (red triangle scheme) compared to the control group (blue circle scheme), as observed by radio telemetry. Numbers indicate sample sizes. Sample sizes for every section decreased stepwise as not every trait could be assigned for every bird (see methods for details). (*a*) Departure probability as proportion of birds departing on the first night after the treatment from Helgoland (white numbers, lower bar) or staying for the first night (black numbers, upper bar). (*b*) Departure timing as minutes after sunset. Asterisk: one juvenile control and one adult experimental bird departed before sunset (−167 and −235 min after sunset, respectively). (*c*) Initial departure direction from Helgoland. (*d*) The consistency of flight direction after departure from Helgoland until passage at the coastline (50–100 km), given as the directional deviation between departure direction from Helgoland and passage location site on the coastline (see methods for details). Dashed lines in circular plots indicate 95% confidence intervals. Data points in the circular plots are shifted slightly off-centre by less than 5° between the groups to better distinguish the data of the corresponding groups. Photos by T.K.

In the control group, 28 out of 33 juvenile wheatears departed on the first night (five birds stayed for 1 day), while all 35 adult wheatears departed on the first night (figure 3*a*). In the experimental group, 32 out of 35 juvenile wheatears departed on the first night (two birds stayed for 1 day and one bird for 5 days), whereas 33 out of 34 adult wheatears left the island in the first night (figure 3*a*). We found no significant differences in the departure probability during the first night after the yes/no pulse application for juvenile (Pearson's *χ*^2^-test: *χ*^2^_1_ = 0.216, *p* = 0.642) and adult wheatears (Pearson's *χ*^2^-test: *χ*^2^_1_ < 0.001, *p* = 0.988).

### 3.2. Departure timing within the night

Juvenile control wheatears departed 83 min after sunset (median; 1st quartile: 67 min; 3rd quartile: 104 min; range: −167 to 207 min; *n* = 33) from Helgoland and juvenile experimental wheatears after 79 min (median; 1st quartile: 64 min; 3rd quartile: 101 min; range: 51 to 352 min; *n* = 33). Adult control wheatears left Helgoland 76 min after sunset (median; 1st quartile: 67 min; 3rd quartile: 92 min; range: 45 to 458 min; *n* = 35) and adult experimental wheatears after 71 min (median; 1st quartile: 61 min; 3rd quartile: 84 min; range: −235 to 248 min; *n* = 34) (figure 3*b*). We did not find a significant effect of treatment group on the bird's departure timing within the night (*p* = 0.349; electronic supplementary material, table S2). Fuel load had a significant negative effect on departure timing, meaning that birds with a higher fuel load departed earlier in the night (electronic supplementary material, table S2), but fuel load between treatment groups did not differ (electronic supplementary material, table S1).

### 3.3. Departure direction

Departure directions from Helgoland of all groups were significantly oriented southwards (figure 3*c*). Juvenile birds in the control group departed, on average, towards 154° (Rayleigh test *r* = 0.781, *p* < 0.001, *n* = 29), and pulse-treated juvenile birds towards 161° (Rayleigh test *r* = 0.743, *p* < 0.001, *n* = 29). Adult birds of the control group departed, on average, towards 167° (Rayleigh test *r* = 0.741, *p* < 0.001, *n* = 33), and pulse-treated adults towards 175° (Rayleigh test *r* = 0.718, *p* < 0.001, *n* = 33). In both age groups, we did not find a significant difference between the treatment groups in mean direction or angular variance (Mardia–Watson–Wheeler test: juveniles: *W* = 0.614, d.f. = 2, *p* = 0.736; adults: *W* = 0.400, d.f. = 2, *p* = 0.819).

### 3.4. Consistency in flight direction

All groups kept the direction in which they departed from Helgoland until they reached the coastline after about 50–100 km flight with an accuracy of about -10° anticlockwise (=10° clockwise; figures 1*b*,*d*,3*d*). Juvenile birds in the control group shifted their flight direction on average by −13° (Rayleigh test *r* = 0.852, *p* < 0.001, *n* = 25) and the experimental juveniles by −15° (Rayleigh test *r* = 0.888, *p* < 0.001, *n* = 21). Adult birds of the control group altered flight direction by −5° (Rayleigh test *r* = 0.873, *p* < 0.001, *n* = 27) and the experimental adults by −8° (Rayleigh test *r* = 0.873, *p* < 0.001, *n* = 27). We did not find a significant effect of the magnetic pulse on the consistency of the flight direction in juvenile or adult birds (Watson–Williams test, juveniles: *F*_1,44_ = 0.087, *p* = 0.769; adults: *F*_1,52_ = 0.149, *p* = 0.701).

## 4. Discussion

In agreement with our predictions for juvenile wheatears, we did not find any differences in the four migratory traits, i.e. departure probability, departure timing, departure direction and consistency in flight direction, between control and experimental, i.e. magnetic pulse treated birds. Unexpectedly, we also did not find any significant effect of the magnetic pulse on the migratory traits in adult wheatears (figure 3). This lack of effect is in stark contrast with our predictions, previous studies and current knowledge about the magnetic-particle-based mechanism. These results open the discussion in two main directions: first, but less likely, the magnetic-particle-based sensor was not affected by the magnetic pulse or does not even exist. Second, the magnetic-particle-based sensor was affected, but the birds did not show any response to the treatment.

### 4.1. Magnetic-particle-based sensor not affected

Beyond the inclination compass, there is good experimental evidence on songbirds for the existence of a second magnetoreception system, used for extracting magnetic information relevant for the navigational map [9,10,12,15,16]. In wheatears in particular, Bulte *et al*. [64] demonstrated that captive-bred birds experiencing virtually changing geomagnetic cues lowered the extent of their migratory restlessness when ‘approaching' their migratory destination. Moreover, Elbers *et al*. [65] observed an increased activation of the trigeminal brainstem in wheatears experiencing artificial magnetic stimuli. Likewise, ablation studies showed that the ophthalmic branch of the trigeminal nerve is necessary for magnetic map navigation [66,67], although not at all release sites [68]. Because the ophthalmic branch conveys sensory information from the upper beak to the trigeminal brainstem, the putative magnetic-particle-based sensors are probably located in nerve endings in the upper beak, the central target of our pulse. A putative magnetoreceptor based on Faraday induction in the semicircular canals, as proposed for homing pigeons [69], would also be exposed to the pulse (figure 2*b*), but the induced voltage spike would amount to less than 10 mV, given that we measured 3 V with a *ca* 500 times larger pick-up coil area compared to the area of a semicircular canal. A pulse is therefore unlikely to impair this mechanism, which is not supported in songbirds by evidence either.

By contrast, the magnetic pulse was strong enough to impair or to misadjust a magnetite-based sensor, be it disruption of interacting clusters of superparamagnetic particles [22], or be it remagnetisation of single-domain particles. Pulse-field remagnetisation studies on magnetotactic bacteria yielded typical switching fields of 30 mT, with 82 mT being the maximum reported [70]. The intensity of our magnetic pulse measured at the position of the beak, 100 mT, was identical to that used in Holland [36] and Holland & Helm [37]. In both of their studies, the pulse yielded the expected response. Likewise, bats with a hypothesised magnetic-particle-based compass sensor were affected by a pulse of 100 mT intensity [71], whereas the orientation of homing pigeons was unaffected [72].

Last, we consider the potential role of the pulsing geometry. In almost all pulsing experiments, the head of the birds was pointing along the pulse coil axis so that the pulse can be expected to affect both hemispheres equally. This practice was introduced in the early studies [23,24,28] using a solenoid with nearly 10 cm clear inner diameter, which invites placement of the bird with the head forward in. We decided to pulse the birds in perpendicular orientation (figure 2*b*), which breaks the bilateral symmetry, with the intention to produce some bias which would result in a more pronounced deflection. Indeed, in an earlier study on homing pigeons, it was found that on the day of treatment, a perpendicular pulse caused a significantly larger deflection than an axial pulse [73]. While different pulsing geometries have been found to produce different deflections [37,73], the very fact that our birds did not seem to be affected by a pulse at all suggests that the role of the pulse geometry is subordinate compared to the navigation strategy at the release site, which we will discuss below.

### 4.2. Magnetic-particle-based sensor affected

Ample evidence [18,23,27,36,37] strongly suggests the effect of a magnetic pulse on navigation behaviour, possibly mediated by affecting a magnetic-particle-based sensor used for geomagnetic map sensing. However, the lack of information about the sensor makes it difficult to estimate how a magnetic pulse might affect reception and with it, the internal representation of the geomagnetic map, which probably consists of a combination of inclination, intensity and declination [18]. Any change in the internal representation after magnetic pulsing could translocate the bird to another known location on its geomagnetic map, similar to virtual magnetic translocation [12,15,16], or provide conflicting and thus unexpected geomagnetic information. Even in the case of magnetic translocation, we would only expect to find an effect if the magnetic pulse ‘translocated’ the birds sufficiently far to the east or west. Short or even north/south translocations may not necessarily lead to a significant change in the departure direction, as the migratory destination is about 4500 km to the south of Helgoland. For example, a translocation to the west from Helgoland to Ireland, i.e. 1000 km, does not substantially change the vector direction towards the wintering grounds in sub-Sahelian western Africa. Under such circumstances, adult wheatears would not necessarily have to compensate for the effect of the magnetic pulse in terms of temporal and directional departure decisions. If the effect instead is an ‘unrealistic' combination of geomagnetic map cues, i.e. a location not compatible with a bird's map, or makes the magnetic sense unreadable, our birds may have ignored the ambiguous map information and prioritised clock-and-compass orientation to determine the future departure direction from Helgoland [15,68]. Such a back-up mechanism, i.e. falling back on clock-and-compass orientation when geomagnetic map information is ambiguous, might explain the lack of directional change in our experiment (figure 3*c*). Selection might have favoured this decision pathway as there are also natural situations in which geomagnetic cues are ambiguous: ‘*highly localised and irregularly scattered*' [74, pp. 63-64] magnetic anomalies that occur in places with special geological features in the Earth's crust [11]. Magnetic storms can also disturb the natural field, but occur rarely and timely limited for a maximum of 1 day [11].

If the magnetic pulse ‘virtually translocated' a bird, the related follow-up questions are (i) when does a bird perceive the map information, at the beginning of the stopover and thus before the pulsing or briefly before departure? and (ii) when is this information incorporated into a bird's temporal and directional departure decisions? If both (i) and (ii) occurred in our birds before the treatment was applied, this would explain the lack of an effect (figure 3). In our opinion, this seems unlikely because a recent stopover study strongly suggested that the decision to resume migration is most likely made only a few hours before sunset on the day of departure [41]. Because we applied a pulse to the birds 6 h before sunset, they probably incorporated geomagnetic information in their departure decisions after the treatment. Given the previous work [23,33] showing that birds had taken about 10 days after pulsing to restore their original magnetic orientation directions, we deem it unlikely that the birds recalibrated their magnetic input in as little as 6 h before departing.

However, where and when along the migration route wheatears include geomagnetic map information for their departure decision is unknown, especially since the accuracy of navigation with geomagnetic maps does not work equally well in the world and seems to be rather low at our study side [75]. Currently, we assume that there are three different phases of navigation: the long-distance phase, the homing phase and the pinpointing-the-goal phase [35]. Magnetic map information is supposed to be most important in the former two phases, and far less in the latter [35]. Wheatears on Helgoland during autumn migration are still about 4500 km away from their wintering grounds [40] and thus in the long-distance phase. Despite the predicted importance and usage of geomagnetic cues [35], wheatears might predominantly rely on clock-and-compass orientation during the long-distance phase. This speculation is supported by the predictions [76] and observations [77] that migrant birds should allow for wind drift when still far away from the migratory destination. If so, this tolerance to drifts might explain why we did not find any effect of the magnetic pulse on all the migratory traits in our study. Alternatively, adult birds might also rely on former experience. Because the German Bight is a common stopover site, birds might potentially remember landmarks or other cues and might therefore prioritise such cues over corrupt magnetic map information. However, this seems unlikely as the adults responded similarly to the juveniles to the magnetic pulse and the stopover site fidelity of passerines on Helgoland is extremely low [78]. This prompts the question if European robins and reed warblers from previous studies [36,37] were caught in a critical navigation phase where the pulse treatment was able to affect their departure directions. Both species breed and European robins winter in the surrounding region of their study site in southern Germany [79], so it is probable that at least some birds were in the homing phase, during which the geomagnetic map could play an important role in guiding birds towards their migratory goal [35]. This would explain why there was a stronger effect of the magnetic pulse in these studies [36,37] than in ours. We conclude that detailed knowledge about the ecological background of the migrant birds, especially in relation to the navigational phase and therewith the remaining distance to the goal, has significant biological implications for the cautious interpretation of such navigation experiments.

## 5. Conclusion

By finding no effect of a magnetic pulse on the migration behaviour of free-flying juvenile and adult wheatears, we cannot give any support to the existence and use of the magnetic-particle-based sensor and geomagnetic map for navigation. Despite evidence for the use of geomagnetic cues in wheatears [64,65], other cues here evidently suffice to determine the seasonally appropriate migratory direction from Helgoland when both pulsed and untreated wheatears navigated with apparently the same precision and accuracy (figure 3*c*,*d*). This interpretation is irrespective of whether or not pulsed wheat-ears were still able to detect geomagnetic map cues. Having found no pulse effect on other migration parameters either, we wonder within which ecological context, e.g. navigation phase (long-distance versus homing phase) or migration strategy (long- versus short-distance migrants), and for which migratory decisions, songbirds might use a geomagnetic map.

## Data accessibility

The data are provided in the electronic supplementary material.
